# The Preyssler-Type Polyoxotungstate Exhibits Anti-Quorum Sensing, Antibiofilm, and Antiviral Activities

**DOI:** 10.3390/biology11070994

**Published:** 2022-06-30

**Authors:** Leonor Faleiro, Ana Marques, João Martins, Luísa Jordão, Isabel Nogueira, Nadiia I. Gumerova, Annette Rompel, Manuel Aureliano

**Affiliations:** 1 Faculdade de Ciências e Tecnologia, Universidade do Algarve, Campus de Gambelas, 8005-139 Faro, Portugal; a54358@ualg.pt (A.M.); joao.m4rtins@gmail.com (J.M.); 2 Algarve Biomedical Center—Research Institute, 8005-139 Faro, Portugal; 3 Champalimaud Research Program, Champalimaud Centre for the Unknown, 1400-038 Lisbon, Portugal; 4 Centro de Ciências do Mar (CCMar), Universidade do Algarve, 8005-139 Faro, Portugal; 5 Departamento de Saúde Ambiental (DSA), Instituto Nacional de Saúde Doutor Ricardo Jorge (INSA), Unidade de Investigação e Desenvolvimento, 1649-016 Lisboa, Portugal; maria.jordao@insa.min-saude.pt; 6 MicroLab, Instituto Superior Técnico, Avenida Rovisco Pais, 1049-001 Lisboa, Portugal; isabel.nogueira@tecnico.ulisboa.pt; 7 Universität Wien, Fakultät für Chemie, Institut für Biophysikalische Chemie, 1090 Wien, Austria; nadiia.gumerova@univie.ac.at (N.I.G.); annette.rompel@univie.ac.at (A.R.)

**Keywords:** antibacterial drugs, bacterial resistance, antibiotic resistance, Wells-Dawson polyoxometalates, multiresistant, P_5_W_30_, P_2_W_18_

## Abstract

**Simple Summary:**

Besides showing the antibacterial activity of the Preyssler-type polyoxotungstate (POT) P_5_W_30_ with the ability to affect MRSA cells, we demonstrated that P_5_W_30_ also displays other proprieties, such as anti-quorum sensing and antibiofilm. These are biological activities that are reported for a POT for the first time. Quorum sensing and biofilm facilitate the bacterial colonization, antibiotic resistance and persistence in both the environment and host, and its impairment by POTs can greatly contribute to the control of bacterial infections, such as those caused by multiresistant bacteria. Moreover, antiviral activity was also observed using the enterovirus Qβ. NMR stability studies of P_5_W_30_ demonstrate that it remains intact, suggesting its responsibility in the described biological activities. Taken together, our results emphasize the potential biomedical use of POTs, particularly the Preyssler-type POT, to fight antibiotic-resistant MRSA strains and their ability to form biofilm, besides being a promising antiviral agent.

**Abstract:**

The increase in bacterial resistance to antibiotics has led researchers to find new compounds or find combinations between different compounds with potential antibacterial action and with the ability to prevent the development of antibiotic resistance. Polyoxotungstates (POTs) are inorganic clusters that may fulfill that need, either individually or in combination with antibiotics. Herein, we report the ability of the polyoxotungstates (POTs) with Wells-Dawson P_2_W_18_, P_2_W_17_, P_2_W_15_, and Preyssler P_5_W_30_ type structures to differently affect Gram-negative and Gram-positive microorganisms, either susceptible or resistant to antibiotics. The compound P_5_W_30_ showed the highest activity against the majority of the tested bacterial strains in comparison with the other tested POTs (P_2_W_15_, P_2_W_17_ and P_2_W_18_) that did not show inhibition zones for the Gram-negative bacteria, *A. baumanii* I73775, *E. coli* DSM 1077, *E. coli* I73194, *K. pneumoniae* I7092374, and *P. aeruginosa* C46281). Generally, the results evidenced that Gram-positive bacteria are more susceptible to the POTs tested. The compound P_5_W_30_ was the one most active against *S. aureus* ATCC 6538 and MRSA16, reaching <0.83 mg·mL^−1^ (100 μM) and 4.96 mg·mL^−1^ (600 μM), respectively. Moreover, it was verified by NMR spectroscopy that the most promising POT, P_5_W_30_, remains intact under all the experimental conditions, after 24 h at 37 °C. This prompted us to further evaluate the anti-quorum sensing activity of P_5_W_30_ using the biosensor *Chromobacterium violaceum* CV026, as well as its antibiofilm activity both individually and in combination with the antibiotic cefoxitin against the methicillin-resistant *Staphylococcus aureus* 16 (MRSA16). P_5_W_30_ showed a synergistic antibacterial effect with the antibiotic cefoxitin and chloramphenicol against MRSA16. Moreover, the antibiofilm activity of P_5_W_30_ was more pronounced when used individually, in comparison with the combination with the antibiotic cefoxitin. Finally, the antiviral activity of P_5_W_30_ was tested using the coliphage Qβ, showing a dose-dependent response. The maximum inactivation was observed at 750 μM (6.23 mg·mL^−1^). In sum, P_5_W_30_ shows anti-quorum sensing and antibiofilm activities besides being a potent antibacterial agent against *S. aureus* and to exhibit antiviral activities against enteric viruses.

## 1. Introduction

Antibiotic resistance (AR) is an ancient natural process that is based on different mechanisms, mainly associated with (1) enzymatic inactivation or alteration of the antibacterial agent, (2) protection, modification, or replacement of the molecular target, (3) impediment of antibiotic permeation into the cell, and (4) active efflux pump from the cell [1,2]. The misuse of antibiotics in both human therapy and veterinary medicine allowed this natural process to evolve at an ever-increasing rate, thereby jeopardizing the control of severe bacterial infections [2]. AR, due to its prevalence in the environment and in food, is not only a problem in clinics [3]. In 2017, the most serious bacterial pathogens associated with health care infections (HCI) were classified by the World Health Organization (https://www.who.int/news/item/27-02-2017-who-publishes-list-of-bacteria-for-which-new-antibiotics-are-urgently-needed, accessed on 17 December 2021) into three groups of priority, critical, high, and medium, for the development of new antibiotics to control the infections caused by these bacterial pathogens. In the group of critical and high-priority ones are the designated ESKAPE bacterial pathogens (*Enterococcus faecium* vancomycin-resistant [VRE], *Staphylococcus aureus*, methicillin-resistant, vancomycin-intermediate and resistant [MRSA/VRSA], *Klebsiella pneumoniae*, *Acinetobacter baumanii*, *Pseudomonas aeruginosa* and *Enterobacteriaceae* carbapenem-resistant and third-generation cephalosporin-resistant). The ESKAPE pathogens are deadly, infectious bacteria that are brilliant at developing multiresistant patterns. Apart from the fact that they are genetically different, their mechanisms of resistance consist of the previously mentioned antibiotic target change, inactivation, decreased uptake, and drug efflux pumps [2]. The ability of pathogens to adhere to biotic or abiotic surfaces is a driving force in their antibiotic resistance and virulence [4,5]. The process of biofilm formation is established by a series of events, namely the initial adhesion, permanent adhesion, production of exopolysaccharides that confer a spatial structure, biofilm maturation, and disaggregation [6]. The protection of sessile cells (adherent) against antimicrobial agents, including phagocyte attack, is guaranteed by the exopolysaccharide matrix [7,8].

Biofilms are a source of contamination of medical devices, such as prosthetic joint, urinary catheters, central venous catheters, among others, and are of great concern in health care [9]. Biofilm formation, expression of virulence factors, toxin production, and antibiotic resistance are regulated by the chemical cell–cell communication system denominated by quorum sensing (QS). The QS system functions in coordination with cell density, permitting the adaptation of the microbial cells to environmental changes involving the production and detection of secreted signaling molecules designated by autoinducers [10]. The use of QS is common in both Gram-positive and Gram-negative bacteria. However, their signaling molecules are different. In Gram-negative bacteria, the autoinducer molecules are acyl-homoserine lactones (AHLs) or other molecules that are produced from the enzymatic cofactor S-adenosylmethionine (SAM), whereas the autoinducers in Gram-positive bacteria are peptides [10,11]. Autoinducers freely pass the bacterial membrane and the process continues with them attaching themselves to their specific membrane or cytoplasmic receptor, initiating a change in gene expression in a multitude (from dozen to hundreds) of genes that aid critical physiological processes [4,12].

**Figure 1:**
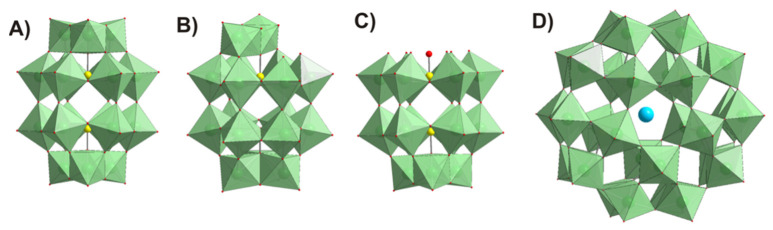
Polyhedral representation of POT structures tested in this study: (**A**) intact Wells-Dawson anion [α-P^V^_2_W^VI^_18_O_62_]^6−^ P_2_W_18_; (**B**) mono-lacunary Wells-Dawson anion [α_2_-P^V^_2_W^VI^_17_O_61_]^10−^ P_2_W_17_; (**C**) tri-lacunary Wells-Dawson anion [P^V^_2_W^VI^_15_O_56_]^12−^ P_2_W_15_; (**D**) Preyssler anion [NaP^V^_5_W^VI^_30_O_110_]^14−^ P_5_W_30_. Color code: {WO_6_}, green; P, yellow; O, red; Na, cyan.

Four main mechanisms of quorum quenching can be highlighted: (1) the inhibition of signal molecule synthesis, (2) inactivation or enzymatic degradation of signal molecules, (3) competition with signal molecules–receptor analogues, and (4) prevention of the signal transduction cascades [13]. The fact that quorum-quenching molecules do not affect the bacterial growth means that the risk of these molecules to induce resistance is minor. Due to the importance of QS in the bacterial pathogenesis process, it becomes an ideal target to control bacterial pathogens with an increased interest to identify QS inhibitors (quorum quenching) [12,13,14]. Several QS inhibitors have been identified, and among them, different phytochemicals [15,16], bacteriophages [17,18], and metal nanoparticles [19,20,21]. The efforts to find compounds able to break the development of antibiotic resistance led to the investigation of metal oxides as antibacterial agents [22,23,24,25,26,27,28]. These metal oxides can easily interact with the bacterial cell membrane due to their small size and active surface ligands, forcing the hyperpolarization of the bacterial cells, and at the same time, immobilizing Mg^2+^ transporters, disturbing the operation of ribosomes, which leads to the disruption of cellular viability without inducing antibiotic resistance [29]. Classic polyoxometalates (POMs) are a well-known group of anionic polynuclear metal oxides (containing V^V^, Ta^V^, Nb^V^, W^VI^ and Mo^VI^ usually in their highest oxidation state) with distinct and chemically changeable cluster structures [30,31,32]. Besides, POMs can include non-metal ions (e. g. P^V^, As^V^, Si^IV^) and one or more of the addenda metal oxo fragments may be absent and/or substituted by other 3d- (e.g., Fe^III^, Co^II^ Ni^II^) or 4f-metal ions (e.g., Ce^III^, Nd^III^, Gd^III^). Due to their structural diversity, POMs have shown specific physicochemical properties responsible for discrete chemical and biological applications, such as catalysis [33], protein crystallization [34], anticancer [35], antibacterial [36,37], and antidiabetic activities [38,39], among others. Phosphotungstates, and especially the doughnut-shaped Preyssler [X^n+^P_5_W_30_O_110_]^(15−n)−^ (X = Na^+^, Ag^+^, Ca^2+^, etc.) anion, have shown great potential as antibacterial [24,29,40,41], antitumor [42,43,44], and antiviral agents [45]. Featured for their ultra-small sizes, favorable thermal and hydrolytic stability, and well-defined surface structures with high affinity to biomacromolecules, phosphotungstates of Dawson and Preyssler archetypes (Figure 1) are explored here for their effect on biological events that contribute to bacterial virulence, such as quorum sensing and biofilm formation. Once the polyoxotungstate (POT) showed powerful antibacterial, QS and antibiofilm activities, the antiviral activity (here P_5_W_30_) was further examined using the enterovirus Qβ.

**Figure 2:**
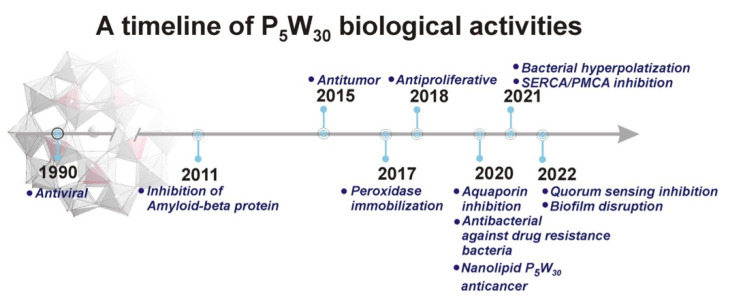
Timeline for P_5_W_30_ biological activities: (1) 1990, Antiviral, (2) 2011, Amyloid beta inhibition; (3) 2015, Antitumor, (4) 2017, Peroxidase immobilization; (5) 2018, Antibacterial; (6) 2020, aquaporin inhibition; antimelanoma activity; (7) 2020, drug-resistance bacteria; (8) Nanolipid-loaded Preyssler for anticancer (9) 2021, bacterial hyperpolarization; (10) 2021, SERCA/PMCA inhibition; (11) 2022, antiquorum sensing; (12) biofilm disruption.

Thus, in addition to the antibacterial and antiviral activities previously described, as well as other biological activities, such as peroxidase immobilization, inhibition of aquaporin, amyloid-beta aggregation, and sarco(endo) reticulum calcium ATPases (SERCA)/plasma membrane calcium ATPases (PMCA) activities [24,29,40,41,42,43,44,45,46,47,48] (Table S1), we show that P_5_W_30_ is also capable to impair quorum sensing and biofilm formation, as illustrated in Table S1 and Figure 2. As can be seen, the first antiviral study was reported in 1990 [45], but the majority of biological investigations have only been reported in the last five years [24,29,40,41,42,43,44,46,49] (Figure 2).

## 2. Materials and Methods

### 2.1. Bacterial Strains and Growth Conditions

The tested microorganisms are indicated in Table 1. All bacterial strains were maintained in Brain Heart Infusion (BHI) (Oxoid, Basingstock, UK), supplemented with glycerol (25% [*v*/*v*]) at −80 °C, except *Chromobacterium violaceum* CV026, which was maintained in Luria Broth Base (LB) (Sigma-Aldrich, St. Louis, MO, USA). The culture media Mueller–Hinton broth (MHB) and Gelose Mueller–Hinton (GMH) were purchased from Biokar Diagnostics (Beauvais). Prior to use, bacteria were transferred to fresh BHI agar plates and incubated at 37 °C. The recovery of *Streptococcus pneumoniae* D39 was performed in BHI supplemented with 5% sheep blood (Oxoid, Basingstock, UK) at 37 °C under microaerophilic conditions using an anaerobic jar.

### 2.2. Polyoxometalates

**Figure 3:**
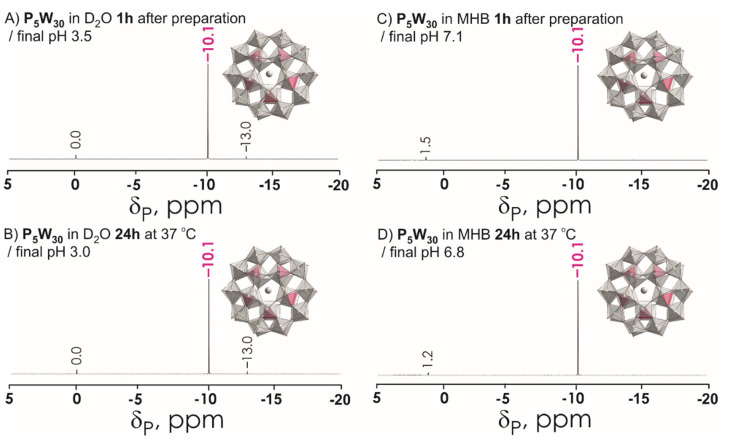
^31^P NMR spectra of P_5_W_30_ 10 mM solutions (**A**) in D_2_O recorded approximately 1 h after preparation; (**B**) in D_2_O recorded after incubation for 24 h at 37 °C; (**C**) in Mueller–Hinton broth (MHB) recorded approximately 1 h after preparation; (**D**) in Mueller–Hinton broth (MHB) recorded after incubation for 24 h at 37 °C. Signals at 0 ppm, 1.2, and 1.5 correspond to free phosphate H*_x_*PO_4_^(3−*x*)−^ (*x* = 0–3). The signal at −10.1 ppm corresponds to 5 equivalent P ions in P_5_W_30_ shown in magenta in polyhedral presentation. Color code: {WO_6_}, light grey; {PO_4_}, magenta; O, red; Na, grey.

**Figure 4:**
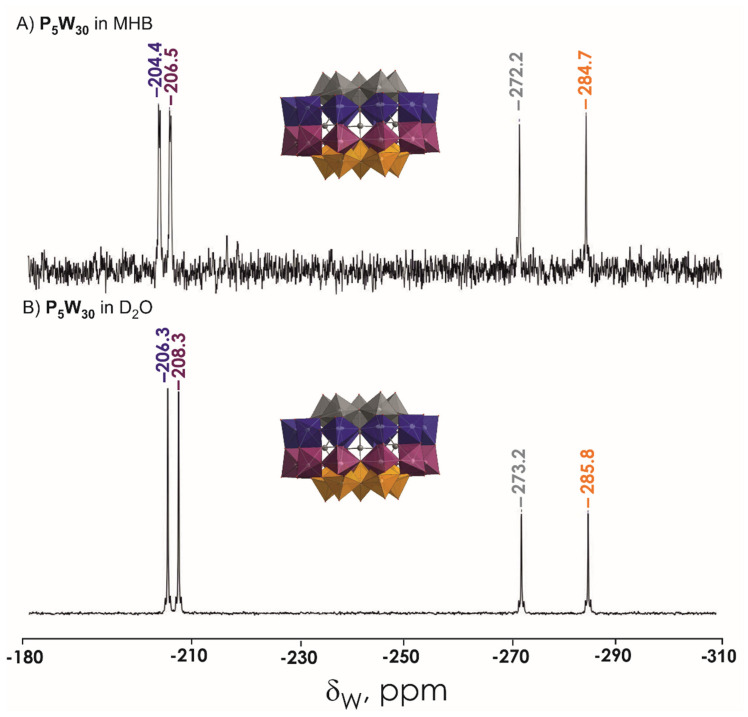
^183^W NMR spectra of 20 mM (NH_4_)_14_[NaP^V^_5_W^VI^_30_O_110_] P_5_W_30_ solution in (**A**) MHB and (**B**) D_2_O. The signals correspond to four types of W ions in P_5_W_30_ shown in different colors. Color code: {WO_6_}, grey, blue, plum, orange; P, grey; O, red.

**Figure 5:**
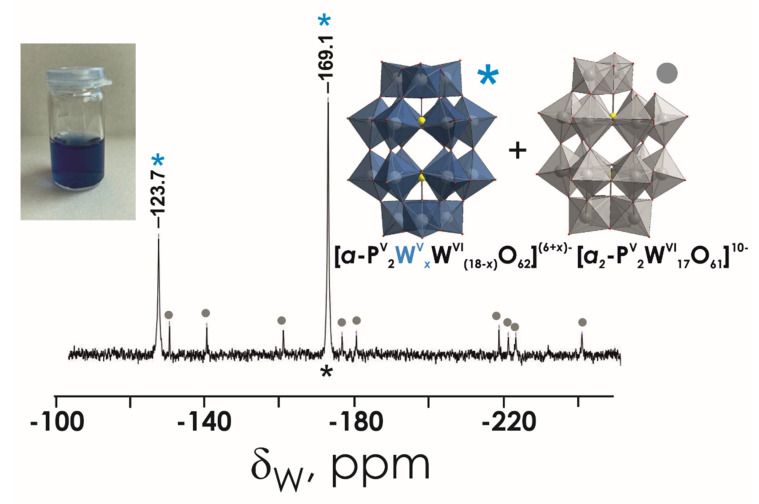
^183^W NMR spectrum of P_2_W_18_ 20 mM solutions in MHB. The signal at −123.7 and −169.1 ppm corresponding to two types of W ions in reduced P_2_W_18_ are depicted with a blue asterisk, and 9 signals of monolacunary P_2_W_17_ are marked with grey circle. Color code: {WO_6_}, grey or blue; P, yellow; O, red.

The POTs used in this study are the intact Wells-Dawson K_6_[*α*-P^V^_2_W^VI^_18_O_62_]·14H_2_O (abbreviated P_2_W_18_) mono-lacunary Wells-Dawson K_10_[*α*_2_-P^V^_2_W^VI^_17_O_61_]·20H_2_O (P_2_W_17_), tri-lacunary Wells-Dawson K_12_[*α-*P^V^_2_W^VI^_15_O_56_]·24H_2_O (P_2_W_15_), and Preyssler (NH_4_)_14_[NaP^V^_5_W^VI^_30_O_110_]·31H_2_O (P_5_W_30_) (Figure 1 and Table 2). The POTs were synthesized according to published procedures [50,51] and their purity was confirmed by infrared, ^31^P and ^183^W NMR spectroscopy (Figure 3, Figure 4, Figure 5 and Figures S1–S4; Table S2). Stock solutions of POTs were freshly prepared by dissolving the solid compound in water and keeping the solution at 4 °C. To determine the antibacterial activity of the POTs, 4 mM solutions of each compound was prepared and subsequently diluted in Mueller–Hinton culture medium to obtain the appropriate final concentrations (Table S3).

#### NMR Spectroscopy

^183^W NMR and ^31^P NMR spectra were recorded with a Bruker FT-NMR spectrometer Avance Neo 500 MHz (Bruker, Rheinstetten, Germany) at 25 °C. Chemical shifts were measured relative to 1 M Na_2_WO_4_ and 85% H_3_PO_4_. ^183^W NMR samples were prepared in 2.7 mL solvent with a POT concentration of 10 mM and measured in 10 mm tubes. The experimental time for ^183^W NMR was ca. 60 h, with a standard pulse program at 20.836 MHz and a 63° flip angle with 1 s relaxation delay. Subsequently, ^31^P NMR spectra were measured at 202.53 MHz in standard 5 mm tubes.

### 2.3. Screening of Antibacterial Activity by Agar Diffusion

Each bacterial strain was previously grown in GMH at 37 °C for 24 h. From this culture a loop was transferred to 10 mL of MHB, and the incubation was done at 37 °C overnight. Afterwards, 8 mL of the previous culture (OD_600nm_ = 0.8–1.0) was transferred into 40 mL of liquefied MH agar medium. The mixture was poured into a sterile Petri dish, which was kept at room temperature, in a laminar flow cabinet, until solidification of the inoculated culture medium. Then, using an inverted sterile Pasteur pipette, 6 mm diameter wells were made into which 40 µL of each compound at the concentrations to be tested were dispensed: for P_5_W_30_, 0.83 mg·mL^-1^ (100 µM), 1.65 mg·mL^-1^ (200 µM), 2.48 mg·mL^-1^ (300 µM), 3.31 mg·mL^-1^ (400 µM), and 4.13 mg·mL^-1^ (500 µM). Incubation was conducted at 37 ± 1 °C for 24 h. The assay with the *S. pneumoniae* D39 bacteria was carried out in Columbia agar (Oxoid, Basingstock, UK) supplemented with 5% sheep blood (Oxoid, Basingstock, UK) and under microaerophilic conditions. Three biological replicates and two technical replicates were performed. After 24 h incubation, the inhibition zone was determined (Table S4).

#### Determination of the Minimum Inhibitory Concentration

The minimum inhibitory concentration (MIC) was determined by microdilution using 96-well flat bottom microplates (Sarstedt Inc, Nümbrecht, Germany). Previously, each bacterial strain was grown in GMH at 37 °C for 24 h. From this culture a loop was transferred to 10 mL of MHB, and the incubation was done at 37 °C overnight. An inoculum density of 5 × 10^5^ CFU·mL^−1^ was used. The total volume in each well was 200 μL and the concentrations tested ranged between 0.44 and 9.82 mg·mL^−1^. The tested concentrations for all POMs were from 100 to 1000 μM. For example, for the compound P_5_W_30,_ the tested concentrations were 0.83 mg·mL^−1^ (100 μM), 1.65 mg·mL^−1^ (200 μM), 3.31 mg·mL^−1^ (400 μM), 4.96 mg·mL^−1^ (600 μM), 6.61 mg·mL^−1^ (800 μM), and 8.26 mg·mL^−1^ (1000 μM). The antibiotic chloramphenicol (30 μg·mL^−1^) was used as control. A set of wells containing only the culture medium was included as the negative control. Three biological and three technical replicates for each strain were used. The incubation of the microplate was performed at 37 °C for 24 h. The bacterial growth was followed by spectrophotometry (OD_600 nm_) in a microplate reader (Tecan Infinite M200, Tecan, Austria). The MIC value was considered the lowest concentration of the compound that caused the inhibition of the bacterial growth (95–100%). The lowest concentration that did not allow the recovery of cells in GMH plates was considered the minimum bactericidal concentration (MBC). The MIC values for the MRSA16 strain for the antibiotics erythromycin, vancomycin, cefoxitin, and chloramphenicol were determined and the resistant profile was established according to the conventional breakpoints [55].

To determine further compound interactions with antibiotics to combat MRSA16 strain, the combinations of P_5_W_30_ with the antibiotics chloramphenicol, cefoxitin, and vancomycin were evaluated using POT concentrations from 100 to 1000 μM. For example, for P_5_W_30_, values were 0.83 mg·mL^−1^ (100 μM), 1.65 mg·mL^−1^ (200 μM), 3.31 mg·mL^−1^ (400 μM), 4.96 mg·mL^−1^ (600 μM), 6.61 mg·mL^−1^ (800 μM), and 8.26 mg·mL^−1^ (1000 μM). The tested concentrations of vancomycin were 0.5 μg·mL^−1^ and 2 μg·mL^−1^ and the concentration of cefoxitin was 2 μg·mL^−1^. The Fractional Inhibitory Concentration (*FIC*) index (Σ*FIC*) was calculated for the combination of compound P_5_W_30_ with the antibiotic against MRSA16 using Equations (1) and (2):

Equation (1):
  (1) $$FIC_{\left(P5W30\right)}=\frac{MIC_{\left(P5W30\;in\;the\;presence\;of\;the\;antibiotic\;\right)}}{MIC_{\left(P5W30\right)}}$$
Equation (2):
  (2) $$FIC_{\left(antibiotic\right)}=\frac{MIC_{\left(antibiotic\;in\;the\;presence\;\mathrm{of}\;P5W30\right)}}{MIC_{\left(antibiotic\right)}}$$
  The Ʃ*FIC* was calculated according to the equation Ʃ*FIC* = *FIC*_(__P5W30)_ + *FIC*_(*antibiotic*)_

The interpretation of Ʃ*FIC* was done according to EUCAST [56].

### 2.4. Inhibition of Quorum Sensing

The anti-quorum-sensing assay was performed in a 96-well flat-bottom microplate (Sarsted Inc, Nümbrecht, Germany) using the biosensor *Chromobacterium violaceum* CV026 [57]. The biosensor was cultivated on LB agar plates at 30 °C for 24 h. A loop from this culture was transferred into 10 mL of LB broth following incubation in a water-bath at 30 °C overnight. A volume of 100 μL of the overnight culture with an optical density (OD_600nm_) of 1.2 was transferred to 100 μL of LB broth, followed by the addition of N-hexanoyl-homoserine lactone (C6-HSL) (Sigma-Aldrich, St. Louis, MO, USA) to a final concentration of 0.2 μg·mL^−1^ in the control (induction of violacein formation) and P_5_W_30_ samples (inhibition of violacein formation). Control wells with no C6-HSL or P_5_W_30_ were included. The tested concentrations of P_5_W_30_ were 100 μM (0.83 mg·mL^−1^), 200 μM (1.65 mg·mL^−1^), 300 μM (2.48 mg·mL^−1^), and 400 μM (3.31 mg·mL^−1^). The incubation was performed at 30 °C over 24 h. After this time interval the inhibition of violacein was evaluated. The optical density of the wells was measured at 585 nm in a microplate reader (Tecan Infinite M200, Tecan, Austria). The assays were performed in triplicate.

For growth control a similar microplate without the addition of C6-HSL was prepared. The antibiotic chloramphenicol (30 μg·mL^−1^) was used as control. The bacterial growth was followed by spectrophotometry (OD_600nm_) in a microplate reader (Tecan Infinite M200, Tecan, Austria). The assays were also performed in triplicate.

### 2.5. Inhibition of Biofilm Formation

The antibiofilm activity of the compound P_5_W_30_ was determined according to the method described by Walker et al. [58] applying slight modifications. Briefly, *Staphylococcus aureus* methicillin-resistant 16 (MRSA16) was grown on BHI agar plates at 37 °C for 24 h. From this culture one isolated colony was transferred into 10 mL of BHI broth and incubated at 37 °C in a water-bath overnight with agitation (120 rpm). Plastic coverslips (unbreakable; 22 × 22 mm; Fisherbrand) were distributed in 6-well flat-bottom plates (Greiner Bio-One GmbH, Kremsmünster, Austria) and sterilized in a flow cabinet for 2 h under ultraviolet light. From the overnight bacterial culture 300 μL was transferred into 2700 μL of BHI broth and this suspension was transferred into each well covering the coverslip. The formation of biofilm was allowed to be produced for 24 h at 37 °C. After biofilm formation, the bacterial culture was eliminated and each well was washed 4 times with phosphate-buffered saline (PBS) to remove non-adherent cells. The biofilm slides were treated with P_5_W_30_ (2× MIC, 10 mg·mL^−1^) and with P_5_W_30_ plus cefoxitin (0.7 mg·mL^−1^ + 2 μg·mL^−1^) for 6 h and 24 h. Non-treated coverslips were used as control. The quantification of the sessile cells (adherent) was conducted by washing each coverslip 4 times with PBS. Each coverslip was transferred into 10 mL of BHI supplemented with 0.05% Tween 80. The tube was sonicated for 7 min at 4 °C. After sonication the coverslip was immediately removed and serial dilutions were prepared by transferring 100 μL of the sonicated culture into 900 μL of PBS. The viable counts were determined by the drop method [59] in BHI agar plates that were incubated at 37 °C for 24 h. Three biological and two technical replicates were used.

### 2.6. Antiviral Activity

The antiviral activity of the compound P_5_W_30_ was evaluated using a microplate dilution method [60] followed by the double-layer agar [61]. The Qβ phage was exposed to P_5_W_30_ at concentrations of 250 μM (2.08 mg·mL^−1^), 500 μM (4.15 mg·mL^−1^), and 750 μM (6.23 mg·mL^−1^) over 24 h. Afterwards, the treated phage suspensions were serially diluted using PBS. For the microplate technique 180 µL of the host bacterial culture in exponential phase (OD_600nm_ = 0.25–0.3) was distributed through a 96-well flat-bottom microplate, and the wells were inoculated with 20 µL of each phage dilution. The bacterial culture in BHI without the compound and the bacterial culture supplemented with the compound at the tested concentrations were used as control. The microplate was incubated at 37 °C and bacterial lysis was monitored every 6 h using a microplate reader (OD_600nm_) (Infinite M200, Tecan, Austria). The lowest two dilutions that show bacterial lysis were selected for quantification of the number of phage particles by the double-layer agar. For this, 90 μL of the phage dilution was transferred into 2 mL of the host bacterium culture (OD_600nm_ = 0.25–0.3) and the contact between the phage and the bacterial host was allowed for 30 min at 37 °C with agitation (120 rpm) in a water-bath. After 30 min of contact, the bacterial and phage suspensions were transferred into 5 mL of semi-solid agar (0.75% (*w*/*v*)), which was then poured onto plates previously prepared with a first layer of solid medium. The inoculated plates were incubated at 37 °C for 24 h. Three biological and three technical replicates were performed.

### 2.7. Transmission Electron Microscopy

The effect of P_5_W_30_ on the MRSA16 cells was analyzed by TEM as previously described by El-Guendouz [62]. For this, 1 mL of bacterial cultures exposed to P_5_W_30_ at concentration of 1211 μM (10 mg·mL^−1^) and P_5_W_30_ plus cefoxitin 85 μM + 400 μM (0. 7 mg·mL^−1^ + 2 μg·mL^−1^) was centrifuged for 10 min at 12,000× *g* at 4 °C. Afterwards, the bacterial cells were resuspended in a mixture of fixatives composed of 2.5% (*v*/*v*) glutaraldehyde and 4% formaldehyde (*w*/*v*) in PBS, maintained at room temperature and protected from light for 2 h. The fixatives were eliminated by centrifugation at 12,000× *g* for 20 min and the bacterial cells were resuspended in 1% (*w*/*v*) paraformaldehyde and maintained at 4 °C until further processing. The fixative was removed by washing with PBS. Bacterial pellets were resuspended in 10% *w*/*v* gelatin (Sigma, St. Louis, MO, USA) in PBS and kept on ice until the solidification of gelatin and small cubes could be cut. The samples were post-fixed by incubation on ice for 2 h in the dark with 1% osmic acid anhydride (OsO_4_) (EMS, Hatfield, PA, USA) in PBS, washed twice with PBS and twice with water before dehydration. Dehydration was accomplished by incubation for 10 min at room temperature 50%, 70%, 96%, and 100% ethanol (Merck, Darmstadt, Germany). The sample was momentarily washed with Epon 812 (EMS) and incubated overnight in the same resin at room temperature. Samples were mounted in molds (EMS) and the Epon 812 was allowed to polymerize in an incubator at 65 °C. Ultra-thin sections were obtained using an ultra-microtome (Leica ultracut R, Leica, Wetzlar, Germany) contrasted with saturated uranyl acetate (Merck, Darmstadt, Germany) in water for 15 min followed by Reynolds lead citrate (Merck, Darmstadt, Germany) for 3 min. Bacterial morphology examined by TEM was performed with a Hitachi H8100 (Hitachi High-Technologies Corporation, Tokyo, Japan). Digital images were acquired using a bottom-mounted CCD Keen-View camera (Olympus Soft Imaging Solutions GmbH, Munich, Germany).

### 2.8. Statistical Analysis

The data were analyzed for statistical significance by one ANOVA using GraphPad Prism (version 9.0) (GraphPad Software, San Diego, CA, USA, www.graphpad.com, accessed on 17 December 2021). Statistical significance was considered at *p* < 0.05; when the analysis was statistically significant, Tukey’s post-hoc test was performed.

## 3. Results and Discussion

### 3.1. Stability Studies

The stability of POTs was tested in water and in MHB by ^31^P and ^183^W NMR spectroscopy. The aqueous solutions of P_5_W_30_, P_2_W_18_, and P_2_W_17_ contain the initial anions [NaP^V^_5_W^VI^_30_O_110_]^14−^, [α-P^V^_2_W^VI^_18_O_62_]^6−^, and [α_2_-P^V^_2_W^VI^_17_O_61_]^10−^, respectively, even after 24 h incubation at 37 °C (Figure 3A,B and Figures S2–S4). Regarding P_2_W_15_ (Figure S4), after dissolution in water, it immediately rearranged to P_2_W_17_, which is very stable under these conditions [63]. In MHB medium, which was used for antibacterial studies, P_2_W_18_ partially (around 30% based on the integration of ^31^P signals) hydrolyses to the monolacunary anion [α_2_-P^V^_2_W^VI^_17_O_61_]^10-^ (Figure 4 and Figure S2C,D). Conversely, Preyssler POT is still the only species present in MHB (Figure 2C,D and Figure 3A,B), which is consistent with previous reports [29,41], indicating the stability of this anion under physiological conditions. In MHB solutions of P_2_W_17_ and P_2_W_15,_ before and after incubation, the predominant (100% for P_2_W_17_ and 97% for P_2_W_15_) POT is [α_2_-P^V^_2_W^VI^_17_O_61_]^10−^. It is worth noting that solutions of P_2_W_18_ (Figure 5) and P_5_W_30_ turned blue after dissolution in MHB, indicating a reduction in W^VI^ ions.

### 3.2. Antibacterial Studies

In order to assess the potential antibacterial activity of POTs, we first screened the antibacterial activity of the four POTs, the intact Wells-Dawson K_6_[*α*-P^V^_2_W^VI^_18_O_62_]·14H_2_O (P_2_W_18_), mono-lacunary Wells-Dawson K_10_[*α*_2_-P^V^_2_W^VI^_17_O_61_]·20H_2_O (P_2_W_17_), tri-lacunary Wells-Dawson K_12_[*α-*P^V^_2_W^VI^_15_O_56_]·24H_2_O (P_2_W_15_), and Preyssler-type (NH_4_)_14_[NaP^V^_5_W^VI^_30_O_110_]·31H_2_O (P_5_W_30_), on different Gram-negative and Gram-positive strains, either susceptible or resistant to antibiotics (Table S4). The antibacterial activity was examined using the agar diffusion technique and the MIC values of all polyoxotungstates were determined by microdilution (Table 3). The observed inhibition zones produced by the tested compounds using the agar diffusion technique are summarized in Table S4. The compound P_5_W_30_ showed the highest activity against the majority of the tested bacterial strains in comparison with the other tested POTs (P_2_W_15_, P_2_W_17_ and P_2_W_18_) that did not show inhibition zones for the Gram-negative bacteria, *A. baumanii* I73775, *E. coli* DSM 1077, *E. coli* I73194, *K. pneumoniae* I7092374, and *P. aeruginosa* C46281). *S. aureus* ATCC 6538 was the most susceptible, followed by *S. pneumoniae* D39 to the four polyoxotungstates tested. No inhibition zone was observed against *E. coli* DSM 1077 by the action of P_5_W_30_, in contrast to the multiresistant strain *E. coli* I73194, which showed a slight susceptibility that increased with the concentration (Table S4). The MIC values of the polyoxotungstates for *S. aureus* ATCC 6538 and two MRSA strains are summarized in Table 3. The compound P_5_W_30_ was more active against *S. aureus* ATCC 6538 and MRSA16, reaching <0.83 and 4.96 mg·mL^−1^, respectively (Table 3). This last strain showed the highest MIC values of 7.01 mg·mL^−1^, 7.87 mg·mL^−1^ and 7.76 mg·mL^−1^ for other three POTs P_2_W_15_, P_2_W_17,_ and P_2_W_18_, respectively (Table 3). The MIC values against all three organisms (Table 3) for thee Wells-Dawson POTs P_2_W_15_, P_2_W_17,_ and P_2_W_18_ are very close. The similar values for P_2_W_15_ and P_2_W_17_ correlate with speciation data, showing that the same monolacunary anion [*α*_2_-P^V^_2_W^VI^_17_O_61_]^10−^ is exclusively present in P_2_W_15_ and P_2_W_17_ solutions (Figures S3 and S4). Although in the MHB solution of P_2_W_18,_ both intact [*α*-P^V^_2_W^VI^_18_O_62_]^6^^−^ and monolacunary [*α*_2_-P^V^_2_W^VI^_17_O_61_]^10−^ anions co-exist (Figure S2), the presence of [*α*-P^V^_2_W^VI^_18_O_62_]^6^^−^ does not affect the antibacterial efficacy compared to P_2_W_15_ and P_2_W_17_.

Our findings, together with those obtained by others [22,24,25,64], highlight the potential use of POMs, including POTs, to combat bacterial infections, even those caused by multiresistant strains. Nevertheless, it is important to emphasize the usefulness of using a significant panel of bacterial strains, as evidenced by the results observed in the current study of MRSA strains, where MRSA16 showed a higher MIC value to the tested P_2_W_15_ (>2×, P_2_W_17_ (>2.5×), and P_2_W_18_ (>2.5×), in comparison with MRSA15, and the lowest MIC value difference was observed for P_5_W_30_ (>1.5×). It is important to highlight that the strain MRSA16 showed resistance to erythromycin that, according to EUCAST [55], also evidences resistance to the antibiotics azithromycin, clarithromycin, and roxithromycin, antibiotics in the macrolide group. This group of antibiotics inhibits the translation process, therefore, inhibiting protein synthesis [64]. The resistance of *S. aureus* to these antibiotics is associated with the modification of the target location of macrolide antibiotics, which is mediated by adenyl-N-methyltransferase erythromycin-resistance methylase (Erm) enzymes, codified by the *erm* genes [64]. Another mechanism of resistance to macrolide antibiotics is linked with *msr* genes that codify for ATP binding cassette (ABC) transporters [65]. MRSA16 also showed resistance to chloramphenicol. This antibiotic acts by binding to the 50S ribosomal subunit, thus, blocking the bacterial protein synthesis, and the most common mechanism of resistance identified in *S. aureus* is through the enzymatic inactivation that is carried out by the enzyme chloramphenicol acetyltransferase [66]. Other mechanisms of resistance to chloramphenicol are the extrusion of the antibiotic through an efflux mechanism driven by the chloramphenicol/florfenicol exporter, the 23S rRNA methyl transferase that is also involved in the resistance to linezolid [66,67]. We are aware of the broad mechanisms of resistance to antibiotics and the difficulty to combat infections caused by such multiresistant strains.

In the study of Gumerova et al. [24], P_5_W_30_ showed a MIC value of 1 μg·mL^−1^ for the respiratory bacterial pathogen *Moraxella catarrhalis* ATCC 2346, whereas against the Gram-positive bacteria *S. aureus,* ATCC 29213 and *Enterococcus faecalis* ATCC 29212 MIC values were increased to 16 μg·mL^−1^ and 8 μg·mL^−1^, respectively. The difference in susceptibility to P_5_W_30_ between the tested *S. aureus* strain in the current study and the *S. aureus* ATCC 29213 tested in [24] is caused by the lower susceptibility of *S. aureus* ATCC 6538. Regarding the susceptibility of *E. coli* strains, the one tested in [24] (*E. coli* ECM 1556) showed no susceptibility to this compound, as observed in the current study for *E. coli* DSM 1077. In contrast, as mentioned, the multiresistant strain *E. coli* I73194 exhibited a very weak susceptibility in the agar diffusion technique.

**Figure 6:**
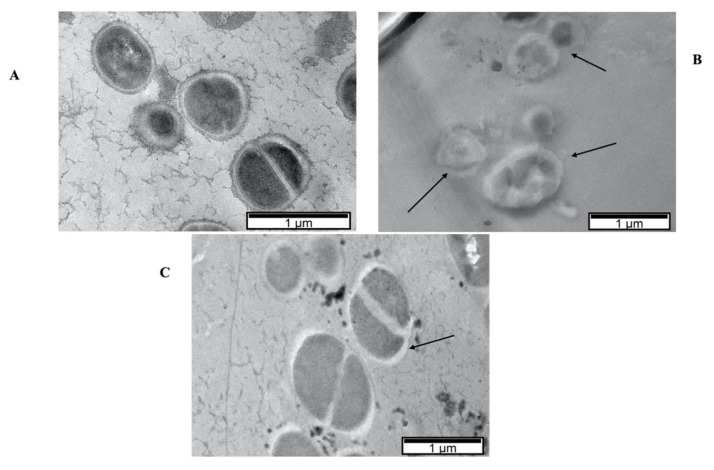
TEM micrographs of MRSA16 cells treated with P_5_W_30_ at 2 × MIC value (1211 μM [10 mg·mL^−1^]) (**B**) and with the combination of P_5_W_30_ with cefoxitin (85 μM + 400 μM [0.7 mg·mL^−1^ + 2 μg·mL^−1^]) (**C**) and control cells (no agent) (**A**). The arrows in (**B**) indicate the severe damage to the bacterial cells caused by the exposure to the compound with loss of cell integrity and in (**C**) the damaged cell wall that will be mainly caused by the combined action of cefoxitin with P_5_W_30_.

The Preyssler-type POT P_5_W_30_ (Figure 1D), which shows the most potent antibacterial activity, was further combined with antibiotics and investigated as an antibiofilm, anti-quorum, and antiviral agent. According to EUCAST recommendations [56], the effect of the combination of P_5_W_30_ with the antibiotic chloramphenicol was additive (ΣFIC = 0.79), whereas for vancomycin, it was indifferent (ΣFIC = 2.0), and for cefoxitin, the observed effect was synergistic (ΣFIC = 0.20). Planktonic cells (in suspension) are known to be more susceptible in comparison with sessile cells (adherent) that are very resistant to the impact of several stress conditions, including antibiotics and other antibacterial agents [15,68]. TEM observations were performed in order to evaluate the impact of the treatment of planktonic MRSA16 cells either with P_5_W_30_ at 2× MIC value (10 mg·mL^−1^), or with P_5_W_30_ (0.7 mg·mL^−1^) combined with cefoxitin (2 μg·mL^−1^). The effect of these treatments on MRSA16 is shown in Figure 6. The MRSA16 cells were severely damaged by P_5_W_30_ at 2× MIC value, showing pronounced shape deformation and loss of cell integrity, resembling protoplasts, in contrast with the bacterial cells treated with the combination of P_5_W_30_ and cefoxitin, which showed mainly a disturbed cell wall (Figure 6). The interaction of P_2_W_18_ with MRSA cell walls has been reported [22], and this interaction occurs with the penetration of the cell wall by the compound leading to its reduction inside the cells (MRSA cells remain blue for about 12 h). The proposed mechanism is associated with the participation of P_2_W_18_ in the electron transfer system for respiration enrolling the NADH/ubiquinone/cytochrome-c, which displays a negative redox potential, able to reduce P_2_W_18_ [22]. Another reported effect of exposure of MRSA strains to P_2_W_18_ is changes at the transcriptome level; namely, the transcripts of *mecA* and *pbp* genes are impaired [22]. We can assume that P_5_W_30_ could act similarly to P_2_W_18_ against MRSA16. Recently, it was described that P_5_W_30_ modulates the cell growth rate via the hyperpolarization of bacterial cells and the so-resulted blocking of the magnesium ion flux into bacterial cells [29]. Moreover, it was described that for both *B. subtilis* and MRSA, the MIC values of P_5_W_30_ in combination with spectinomycin decreased by approximately 10-fold, from about 6–7 mg·mL^−1^ to 0.7 mg·mL^−1^ [29]. These values are in good agreement with the ones obtained in the current study for P_5_W_30_ against MRSA. Taken together, all of these results point to the possibility of realizing the maximum potential of POMs as antibiotics, while mitigating the resistance of pathogenic bacteria.

### 3.3. Anti-Quorum-Sensing Activity

**Figure 7:**
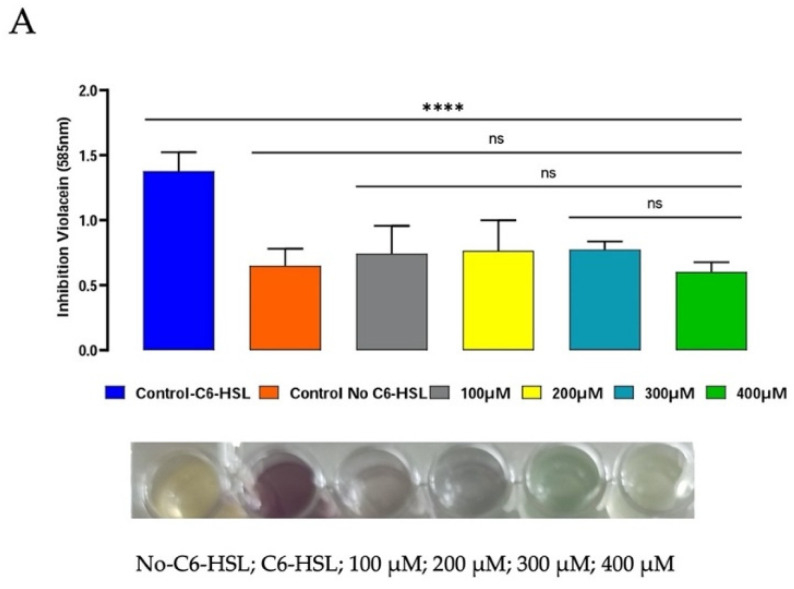

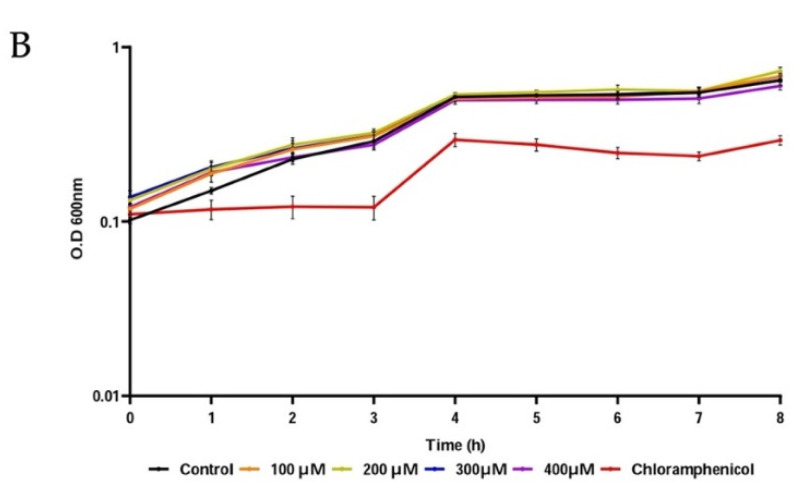
Anti-quorum-sensing activity of P_5_W_30_ at concentrations 100 μM (0.83 mg·mL^−1^), 200 μM (1.65 mg·mL^−1^), 300 μM (2.48 mg·mL^−1^), and 400 μM (3.31 mg·mL^−1^). (**A**) Effect of P_5_W_30_ on violacein production (OD_585nm_). (**B**) Effect of P_5_W_30_ on *C. violaceum* CV026 growth. Error bars represent standard deviation (*n* = 3). **** *p* < 0.0001, ns—not significant.

The anti-quorum-sensing activity of P_5_W_30_ was evaluated using the biosensor *C. violaceum* CV026. The production of violacein by the biosensor strain, the production of which is regulated by the quorum-sensing system [69], was inhibited (*p* < 0.0001) at all concentrations tested: 100 μM [0.83 mg·mL^−1^], 200 μM [1.65 mg·mL^−1^], 300 μM [2.48 mg·mL^−1^], and 400 μM [3.31 mg·mL^−1^] (Figure 7A). No inhibition of the biosensor growth was observed at any tested concentration in the control microplate (no addition of C6-HSL) in comparison with the antibiotic chloramphenicol, for which a 3 h lag phase was observed (Figure 7B). To the best of our knowledge, the current study is the first to report the anti-quorum-sensing activity of a POT compound. As previously mentioned, there are different quorum-quenching mechanisms [13], and future investigations will be of interest to determine the quorum-sensing inhibition mechanisms used by P_5_W_30_ and their impact on reductions in virulence.

### 3.4. Antibiofilm Activity

**Figure 8:**
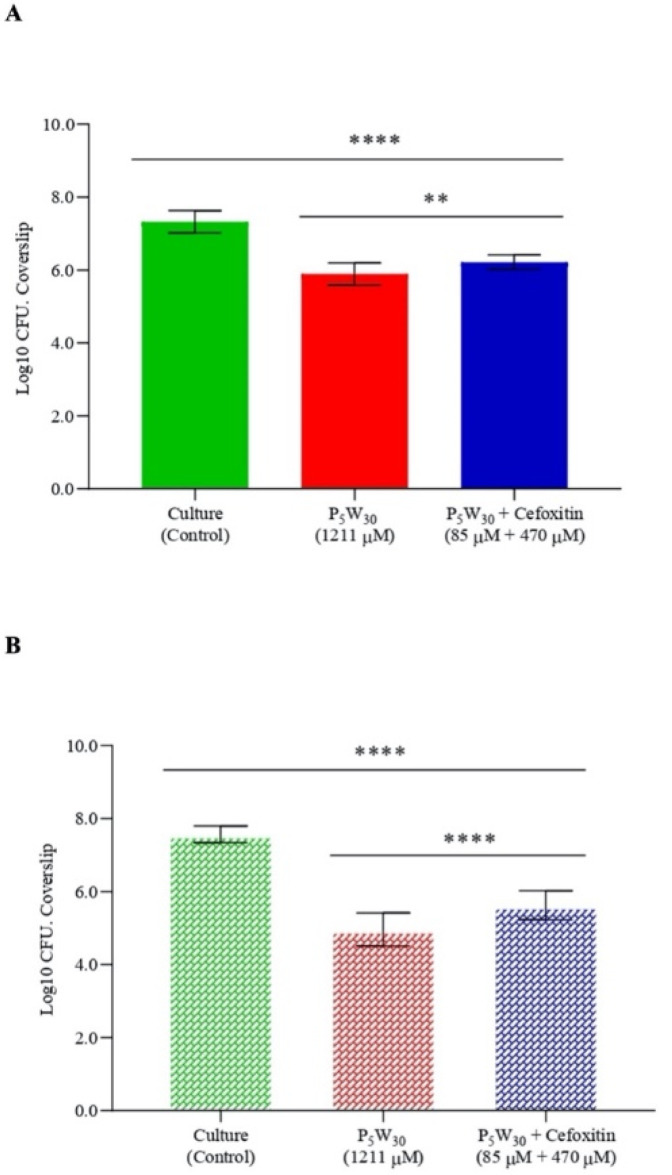
Antibiofilm activity of the compound P_5_W_30_ individually (2× MIC, 1211 μM) and in combination with the antibiotic cefoxitin (85 μM + 470 μM [0.7 mg·mL^−1^ + 2 μg·mL^−1^]) against MRSA16 after 6 h of exposure (**A**) and after 24 h of exposure (**B**). Data are expressed as the mean of three biological and two technical replicates. The error bars represent the standard deviation. **** *p* < 0.0001, ** *p* < 0.05.

For the evaluation of the antibiofilm properties, the MRSA16 cells were allowed to form biofilms in coverslips over 24 h, and afterwards, were treated for 6 h and 24 h with the compound P_5_W_30_ at a concentration of 1211 μM (10 mg·mL^−1^, 2× MIC) and with the combination of P_5_W_30_ with the antibiotic cefoxitin at concentrations of 85 μM + 470 μM (0.7 mg·mL^−1^ + 2 μg^·^mL^−1^). The 2× MIC value of P_5_W_30_ and the combination of P_5_W_30_ with the antibiotic was selected, taking into account the higher resistance of sessile cells that is not affected either by P_5_W_30_ at the MIC value of or by the antibiotic cefoxitin at the MIC value. The results are represented in Figure 8. The disruption of the biofilm produced by MRSA16 was significantly different (*p* < 0.0001) both from the treatment with P_5_W_30_ individually or in combination with the antibiotic cefoxitin after 6 h of treatment. However, the exposure of sessile cells to P_5_W_30_ alone was more detrimental (*p* < 0.05) in comparison with the combination (Figure 8A). The exposure of sessile cells over 24 h to P_5_W_30_ individually or in combination with cefoxitin significantly affected the MRSA16 biofilm (*p* < 0.0001) in comparison with the control (Figure 8B). However, as observed for the 6 h treatment, the exposure of sessile cells for 24 h to P_5_W_30_ alone showed to be more efficient (*p* < 0.001) in the disruption of sessile cells in comparison with the combination (Figure 8B). As expected, the exposure of MRSA16 sessile cells to the concentration of 2× MIC was not sufficient to eliminate these cells; instead, a significant reduction was observed. This result can be explained by the difficulty of P_5_W_30_ to penetrate the matrix of exopolysaccharides that impregnates the aggregated cells in the biofilm. It is possible that the combination of P_5_W_30_ with the antibiotic cefoxitin at concentrations of 85 μM + 470 μM (0.7 mg·mL^−1^ + 2 μg·mL^−1^), although in a more limited way, can disrupt the aggregated cells by interfering with the exopolysaccharide layer. It is important to stress that the antibiotic cefoxitin acts by inhibiting the biosynthesis of the bacterial cell wall by binding to transpeptidases (penicillin-binding proteins, PBPs), and it is resistant to the action of extended-spectrum β-lactamases [70]. Therefore, it is very likely that the binding of the cefoxitin to PBPs will be strongly affected by the exopolysaccharide matrix that protects sessile cells.

As for the anti-quorum-sensing activity, to the best of our knowledge, the current study is the first that describes the antibiofilm action of a POT compound.

### 3.5. Antiviral Activity

**Figure 9:**
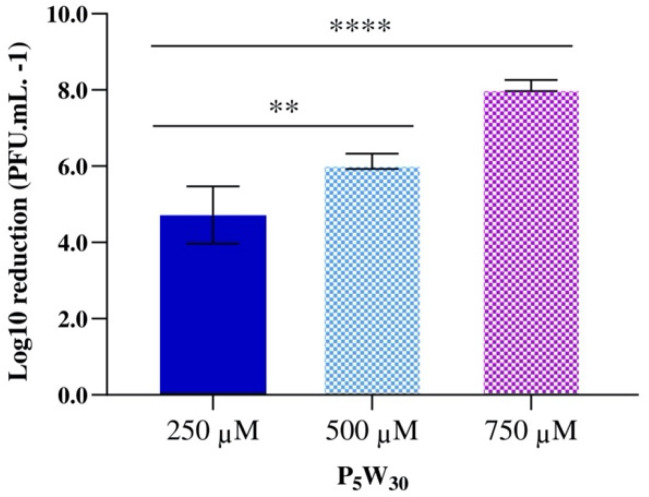
Antiviral activity of the compound P_5_W_30_ against the enterovirus Qβ. Data are expressed as the mean of three biological and three technical replicates. The error bars represent the standard deviation. **** *p* < 0.0001, ** *p* < 0.05.

Finally, the antiviral activity of the compound P_5_W_30_ was evaluated against the enterovirus Qβ at concentrations of 250 μM (2.08 mg·mL^−1^), 500 μM (4.15 mg·mL^−1^), and 750 μM (6.23 mg·mL^−1^). The impact of the compound on the phage infectivity is represented in Figure 9. The reduction in viral particles increased with the concentration of P_5_W_30_ tested. The lowest reduction value (*p* < 0.0001) was observed at a concentration of 250 μM, achieving the maximum reduction at the highest concentration tested of 750 μM (reduction of 8.11± 0.14 Log_10_ PFU·mL^−1^) (Figure 9). The coliphage Qβ of the *Leviviridae* family is a single-stranded, positive-sense RNA phage that is commonly used as a surrogate to mimic the behavior of pathogenic enteric viruses [71]. The ability of POTs to inactivate RNA virus has been reported [47] and the proposed mechanisms of Flu V (influenza virus) inactivation by POTs can occur by inhibiting the virus fusion with the membrane or by inhibiting the binding of the virus to the cell [47]. It is possible that the mechanism of the inactivation of the coliphage Qβ by P_5_W_30_ will be via inhibiting attachment of the phage to the host cells. However, such a hypothesis requires validation.

In sum, in addition to the antibacterial and antiviral activities, we showed that P_5_W_30_ was also able to impair quorum sensing and biofilm formation, as illustrated in Table S1 and Figure 2. Regarding the mode of P_5_W_30_ action and in general for POMs, several potential mechanisms have been evoked and recently summarized [34,35,36,37,39,72], pointing towards promising biological applications in the near future.

## 4. Conclusions

In addition to showing the antibacterial activity of P_5_W_30_ with the ability to affect MRSA cells, we demonstrated that P_5_W_30_ also displays other properties, such as anti-quorum sensing and antibiofilm properties. These are biological activities that are reported for a polyoxotungstate for the first time. Quorum sensing and biofilms facilitate bacterial colonization, antibiotic resistance, and persistence in both the environment and host, and its impairment by POT can greatly contribute to the control of bacterial infections, such as those caused by multiresistant bacteria. Moreover, antiviral activity was also observed using the enterovirus Qβ. NMR stability studies of P_5_W_30_ demonstrate that it remains intact, suggesting that it is an active species in the described biological activities. Taken together, our results emphasize the potential biomedical use of POTs, particularly Preyssler-type, to combat antibiotic-resistant MRSA strains and their ability to form biofilms, in addition to being a promising antiviral agent. The molecular mechanisms of action of the various biological activities described still need to be elucidated, which will lead to a new understanding on a molecular basis.

## Figures and Tables

**Table 1:** Microorganisms used in the study.

Microorganisms	Origin and Characteristics	Source
*Acinetobacter baumannii* DSM 3007	Urine	German Collection of Microorganisms
*Acinetobacter baumannii* I73775	Urine, multiresistant	Laboratory Microbiology, ABC-RI, UAlg ^1^
*Escherichia coli* DSM 1077	K12 *galR arg nad*	German Collection of Microorganisms
*Escherichia coli* DSM 5210	Hfr 3000 U432. Host of phage Qß (DSM 5696)	German Collection of Microorganisms
*Escherichia coli* I73194	Urine, multiresistant	Laboratory Microbiology, ABC-RI, UAlg
*Klebsiella pneumoniae* 70923/4	Urine, multiresistant	Laboratory Microbiology, ABC-RI, UAlg
*Pseudomonas aeruginosa* C46281	Urine, multiresistant	Laboratory Microbiology, ABC-RI, UAlg
*Staphylococcus aureus* ATCC 6538	Wound	American Type Culture Collection
*Staphylococcus aureus methicillin resistant 15 (MRSA15)*	Clinical	Laboratory Microbiology, ABC-RI, UAlg
*Staphylococcus aureus methicillin resistant 16 (MRSA16)*	Clinical	Laboratory Microbiology, ABC-RI, UAlg
*Streptococcus pneumoniae* D39	Clinical	UL, UK ^2^
*Chromobacterium violaceum* (CV026)	(Hg^R^, *cvil*:Tn5 *xyl*E, Kan^R^, higher spontaneous resistance Str^R^)	Gift of Professor Mondher El Jaziri of the University Libre of Brussels

^1^ UAlg—Universidade do Algarve, ABC-Ri-Algarve Biomedical Center—Research Institute, ^2^ UL-University of Leicester, UK.

**Table 2:** POTs used in this study.

Formula	Net Charge in Solid State	Charge Density (Charge/Number of Addenda Atoms Ratio)	First Structural Report in	Synthesis	Anions Present in Aqueous Solution Based on NMR Studies	Anions Present in MHB Based on NMR Studies
K_6_[*α*-P^V^_2_W^VI^_18_O_62_]·14H_2_O (MW 4849.6) P_2_W_18_	−6	0.33	[52]	[50]	[*α*-P^V^_2_W^VI^_18_O_62_]^6−^	[*α*-P^V^_2_W^VI^_18_O_62_]^6−^ + [*α*_2_-P^V^_2_W^VI^_17_O_61_]^10−^
K_10_[*α*_2_-P^V^_2_W^VI^_17_O_61_]·20H_2_O (MW 4914.2) P_2_W_17_	−10	0.56	[47]	[50]	[*α*_2_-P^V^_2_W^VI^_17_O_61_]^10−^	[*α*_2_-P^V^_2_W^VI^_17_O_61_]^10−^
K_12_[*α*-P^V^_2_W^VI^_15_O_56_]·24H_2_O (MW 4617.1) P_2_W_15_	−12	0.80	[53]	[50]	[*α*_2_-P^V^_2_W^VI^_17_O_61_]^10−^	[*α*_2_-P^V^_2_W^VI^_17_O_61_]^10−^
(NH_4_)_14_[NaP^V^_5_W^VI^_30_O_110_]·31H_2_O (MW 8264.0) P_5_W_30_	−14	0.47	[54]	[51]	[NaP^V^_5_W^VI^_30_O_110_]^14−^	[NaP^V^_5_W^VI^_30_O_110_]^14−^

**Table 3:** MIC values for the tested polyoxotungstates (mg·mL^−1^).

Microorganisms	P_2_W_15_	P_2_W_17_	P_2_W_18_	P_5_W_30_
*S. aureus* ATCC 6538	3.54 (800 µM)	2.95 (600 µM)	2.91 (600 µM)	<0.83 (<100 µM)
MRSA15	2.65 (600 µM)	2.95 (600 µM)	2.91 (600 µM)	3.31 (400 µM)
MRSA16	7.01 (1600 µM)	7.87 (1600 µM)	7.76 (1600 µM)	4.96 (600 µM)

## Data Availability

The data presented in the current study are available within the article and Supplementary Material.

## Supplementary Materials

The following supporting information can be downloaded at: https://www.mdpi.com/article/10.3390/biology11070994/s1, Table S1: Biological activities of Preyssler-type POMs, Figure S1: IR spectra of the Wells-Dawson (WD) and Preyssler POTs used in this study, Table S2: Comparison of experimental and reported wavenumbers for P–O vibrations, which are the most important to destinguish between phosphotungstates within one family. Table S3: Experimental conditions and shifts obtained from ^31^P and ^183^W NMR spectroscopic studies performed on the tested POTs, Figure S2: ^31^P NMR spectra of 10 mM K_6_[*α*-P^V^_2_W^VI^_18_O_62_]P_2_W_18_ solutions (A) in D_2_O recorded approximately 1 h after preparation; (B) in D_2_O recorded after incubation for 24 h at 37 °C; (C) in Mueller-Hinton broth (MHB) recorded approximately 1 h after preparation; (D) in Mueller-Hinton broth (MHB) recorded after incubation for 24 h at 37 °C, Figure S3: ^31^P NMR spectra of 10 mM K_10_[*α*_2_-P^V^_2_W^VI^_17_O_61_] P_2_W_17_ solutions (A) in D_2_O recorded approximately 1 h after preparation; (B) in D_2_O recorded after incubation for 24 h at 37 °C; (C) in Mueller-Hinton broth (MHB) recorded approximately 1 h after preparation; D) in Mueller-Hinton broth (MHB) recorded after incubation for 24 h at 37 °C, Figure S4: ^31^P NMR spectra of 10 mM K_12_[P^V^_2_W^VI^_15_O_56_] P_2_W_15_ solutions (A) in D_2_O recorded approximately 1 h after preparation; (B) in D_2_O recorded after incubation for 24 h at 37 °C; (C) in Mueller-Hinton broth (MHB) recorded approximately 1 h after preparation; (D) in Mueller-Hinton broth (MHB) recorded after incubation for 24 h at 37 °C, Table S4: Inhibition zones (mm) produced by the tested polyoxotungstates; K_12_[P^V^_2_W^VI^_15_O_56_] 24H_2_O (P_2_W_15_), K_10_[*α*_2_-P^V^_2_W^VI^_17_O_61_] 20H_2_O (P_2_W_17_), K_6_[*α*-P^V^_2_W^VI^_18_O_62_] 14H_2_O (P_2_W_18_) and (NH_4_)_14_[NaP^V^_5_W^VI^_30_O_110_]·31H_2_O (P_5_W_30_) ranging from 100 to 500 µM.
