# Supplementary material for: The Preyssler-Type Polyoxotungstate Exhibits Anti-Quorum Sensing, Antibiofilm, and Antiviral Activities
Source: Biology (Basel). 2022 Jun 30;11(7):994. doi: 10.3390/biology11070994 (PMC9311568; doi:10.3390/biology11070994)
Supplement: Supplementary file 1 [file biology-11-00994-s001.zip › biology-1750964-supplementary.pdf]

*Supplementary Material*

# The Preyssler-type polyoxotungstate exhibits anti-quorum sensing, antibiofilm, and antiviral activities

**Leonor Faleiro*[1,2,3], Ana Marques[1,2], João Martins[1,4], Luísa Jordão[5], Isabel Nogueira[6], Nadiia I. Gumerova[7], Annette Rompel[7] and Manuel Aureliano*[1,4]**

[1]Universidade do Algarve, Faculdade de Ciências e Tecnologia, C8, Campus de Gambelas, 8005-139 Faro, Portugal
[2]Algarve Biomedical Center - Research Institute, Faro, Portugal
[3]Champalimaud Research Program, Champalimaud Centre for the Unknown, Lisbon, Portugal
[4]Centro de Ciências do Mar (CCMar), Universidade do Algarve, Faro, Portugal
[5]Departamento de Saúde Ambiental (DAS), Instituto Nacional de Saúde Doutor Ricardo Jorge (INSA), Unidade de Investigação e Desenvolvimento, Lisboa, Portugal
[6]MicroLab, Instituto Superior Técnico, Avenida Rovisco Pais, 1049-001 Lisboa, Portugal
[7]Universität Wien, Fakultät für Chemie, Institut für Biophysikalische Chemie, 1090 Wien, Austria; http://www.bpc.univie.ac.at

# Table of content


| | | |
|---|---|---|
| | Biological activities of Preyssler-type POMs | 3 |
| 1 | IR spectroscopy | 4 |
| 2 | NMR Spectroscopy | 6 |
| 2.1 | Preyssler POT | 7 |
| 2.2 | Wells-Dawson POTs | 10 |
| 3 | Inhibition zones | 11 |
| 5 | References | 13 |

# 1 Biological activities of Preyssler-type POMs

**Supplementary Table S1.** Biological activities of Preyssler-type POMs (adapted from [1])

| Preyssler-type POMs | Biological activity | Year | Reference |
|---|---|---|---|
| $(NH_4)_{14}[NaP_5W_{30}O_{110}]$ | Anti-HIV-1 activity, toxicity, and stability | 1990 | 2 |
| $[NaP_5W_{30}O_{110}]^{14-}$ | Inhibition of Amyloid-beta Protein Fibrillization | 2011 | 3 |
| $(CH_3)_{56}N_{14}[(C_{16}H_{33})_2NCONH(CH_2)_3SiNaP_5W_{29}O_{110}]$ | Antitumor activity for human colorectal cancer cell lines (HT29) | 2015 | 4 |
| $(Cu(H_2biim)\}_2)(Na(H_2O)P_5W_{30}O_{110})]\cdot 9\ H_2O\}_n$ | Immobilization of horseradish peroxidase | 2017 | 5 |
| $(NH_4)_{14}[NaP_5W_{30}O_{110}]\cdot 30H_2O$ | Antibacterial activity against *Moraxella catarrhalis* | 2018 | 6 |
| $K_{11}Na_3[NaP_5W_{30}O_{110}]\cdot 29H_2O$<br>$K_{14}[AgP_5W_{30}O_{110}]\cdot 58H_2O\cdot 2KCl$ | Antiproliferation of two human cell lines A431 and HEK293<br>Antibacterial activity against *Escherichia coli* and *Bacillus subtilis* | 2018 | 7 |
| $(NH_4)_{14}[NaP_5W_{30}O_{110}]\cdot 31H_2O$ | Inhibition of aquaporin-3 activity<br>Anti-cancer activity against human melanoma | 2020 | 8 |
| $H_{14}[NaP_5W_{30}O_{110}]$ | In vitro inhibitory effects on HepG2 tumor cells | 2020 | 9 |
| $K_{13}Na[NaP_5W_{30}O_{110}]$ | Antibacterial activity against *Escherichia coli* and *Staphylococcus aureus* | 2020 | 10 |
| $K_{13}Na[NaP_5W_{30}O_{110}]$ | Modulation of bacterial hyperpolarization (*Bacillus subtilis*) | 2021 | 11 |
| $(NH_4)_{14}[NaP_5W_{30}O_{110}]\cdot 31H_2O$ | SERCA/PMCA inhibition | 2021 | 12 |
| $(NH_4)_{14}[NaP_5W_{30}O_{110}]\cdot 31H_2O$ | Antibiofilm and antiquorum sensing activities<br>Antibacterial activity against *S. aureus* ATCC 6538 and MRSA<br>Antiviral activity | 2022 | This study |

# 2 IR spectroscopy

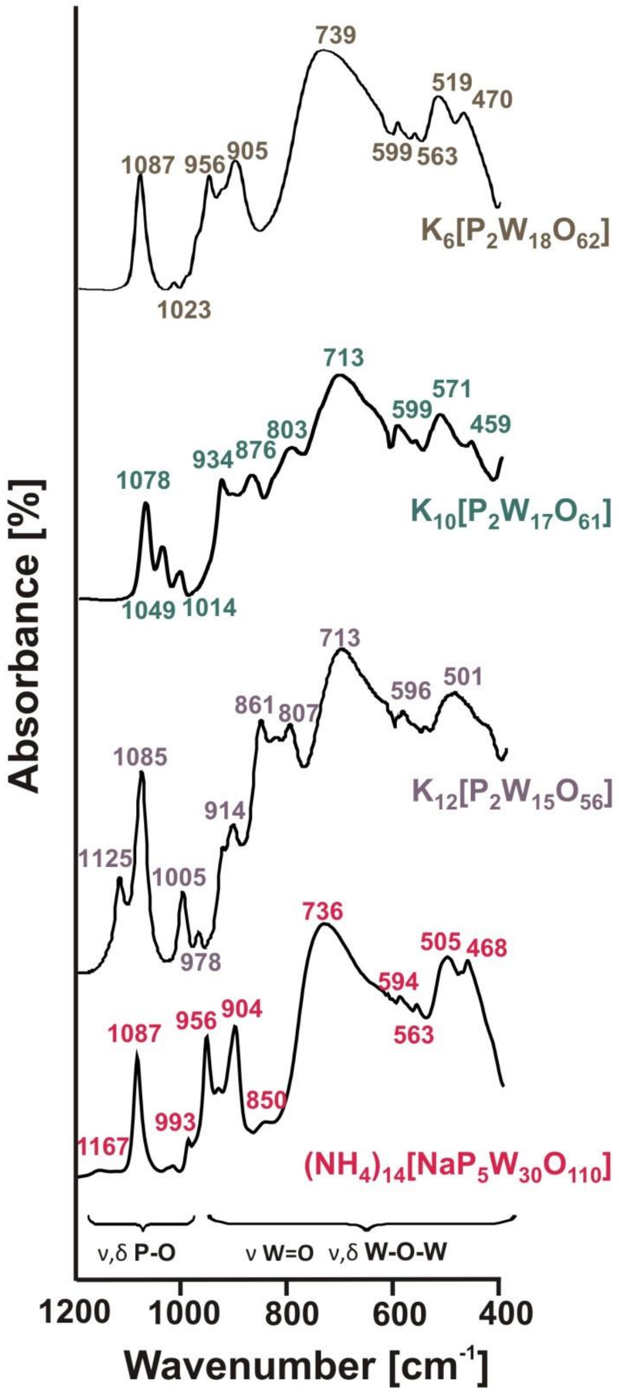


**Supplementary Figure S1.** IR spectra of the Wells-Dawson (WD) and Preyssler POTs used in this study $K_6[\alpha\text{-}P^{V}_2W^{VI}_{18}O_{62}]\cdot14H_2O$ $P_2W_{18,}$ (WD), $K_{10}[\alpha_2\text{-}P^{V}_2W^{VI}_{17}O_{61}]\cdot20H_2O$ $P_2W_{17,}$ (WD), $K_{12}[P^{V}_2W^{VI}_{15}O_{56}]\cdot24H_2O$ $P_2W_{15,}$ (WD), and $(NH_4)_{14}[NaP^{V}_5W^{VI}_{30}O_{110}]\cdot31H_2O$ $P_5W_{30}$ in the region of W-O-W, W-O-P and W=O bond vibrations 1200 – 400 cm$^{-1}$ (see Table S2 for assignment in P-O vibration region).

**Supplementary Table S2.** Comparison of experimental and reported wavenumbers for P–O vibrations, which are the most important to destinguish between phosphotungstates within one family.

| POT | IR band position | |
|---|---|---|
| | **Experimental (Figure S1)** | **Reported in [13] and [14]** |
| $K_6[\alpha\text{-}P^{V}_2W^{VI}_{18}O_{62}]\cdot 14H_2O$ $P_2W_{18}$, (WD) | 1087 and 1023 $cm^{-1}$ | 1087 and 1023 $cm^{-1}$ |
| $K_{10}[\alpha_2\text{-}P^{V}_2W^{VI}_{17}O_{61}]\cdot 20H_2O$ $P_2W_{17}$, (WD) | 1078, 1049 and 1014 $cm^{-1}$ | 1084, 1050 and 1012 $cm^{-1}$ |
| $K_{12}[P^{V}_2W^{VI}_{15}O_{56}]\cdot 24H_2O$ $P_2W_{15}$, (WD) | 1125, 1085 and 1005 $cm^{-1}$ | 1130, 1086 and 1009 $cm^{-1}$ |
| $(NH_4)_{14}[NaP^{V}_5W^{VI}_{30}O_{110}]\cdot 31H_2O$, $P_5W_{30}$ | 1167, 1086 and 1022 $cm^{-1}$ | 1164, 1078 and 1017 $cm^{-1}$ |

**Supplementary Table S3.** Experimental conditions and shifts obtained from $^{31}P$ and $^{183}W$ NMR spectroscopic studies performed on the tested POTs.

| POT | In $D_2O$ | | | | | In $D_2O$ after 24 h at 37 °C | | | | In MHB[a] | | | | | In MHB[a] after 24 h at 37 °C | | | | Ref. |
|---|---|---|---|---|---|---|---|---|---|---|---|---|---|---|---|---|---|---|---|
| | pH | δ $^{31}P$, ppm | δ $^{183}W$, ppm | Species present | Fig. | pH | δ $^{31}P$, ppm | Species present | Fig. | pH | δ $^{31}P$, ppm | δ $^{183}W$, ppm | Species present | Fig. | pH | δ $^{31}P$, ppm | Species present | Fig. | |
| $K_6[\alpha\text{-}P^{V}_2W^{VI}_{18}O_{62}]$ $P_2W_{18}$, (WD) | 5.7 | -13.0 | – | $[\alpha\text{-}P^{V}_2W^{VI}_{18}O_{62}]^{6-}$ | S2A; 4 | 4.6 | -13.0 | $[\alpha\text{-}P^{V}_2W^{VI}_{18}O_{62}]^{6-}$ | S2B | 6.0 | 0.1; -7.3; -13.0; -14.4 | -123.7; -125.0; -137.5; -157.6; -169.1; -172.1, -177.7; -216.0, -218.9; -221.2 | 72%[b] $[\alpha\text{-}P^{V}_2W^{VI}_{18}O_{62}]^{6-}$ + 27%[b] $[\alpha_2\text{-}P^{V}_2W^{VI}_{17}O_{61}]^{10-}$ | S2C; 4 | 4.2 | 0.1; -7.3; -13.0; -14.4 | 69%[b] $[\alpha\text{-}P^{V}_2W^{VI}_{18}O_{62}]^{6-}$ + 29%[b] $[\alpha_2\text{-}P^{V}_2W^{VI}_{17}O_{61}]^{10-}$ | S2D | 13 |
| $K_{10}[\alpha_2\text{-}P^{V}_2W^{VI}_{17}O_{61}]$ $P_2W_{17}$, (WD) | 7.1 | -7.3; -14.4 | – | $[\alpha_2\text{-}P^{V}_2W^{VI}_{17}O_{61}]^{10-}$ | S3A | 6.4 | -7.3; -14.4 | $[\alpha_2\text{-}P^{V}_2W^{VI}_{17}O_{61}]^{10-}$ | S3B | 7.9 | 1.5; -7.3; -14.4 | – | $[\alpha_2\text{-}P^{V}_2W^{VI}_{17}O_{61}]^{10-}$ | S3C | 7.0 | 1.5; -7.3; -14.4 | $[\alpha_2\text{-}P^{V}_2W^{VI}_{17}O_{61}]^{10-}$ | S3D | 13 |
| $K_{12}[P^{V}_2W^{VI}_{15}O_{56}]$ $P_2W_{15}$, (WD) | 9.9 | 2.5; -7.3; -14.4 | – | $[\alpha_2\text{-}P^{V}_2W^{VI}_{17}O_{61}]^{10-}$ | S4A | 7.6 | 2.0; -7.3; -14.4 | $[\alpha_2\text{-}P^{V}_2W^{VI}_{17}O_{61}]^{10-}$ | S4B | 8.5 | 2.1; -7.3; -14.4 | – | $[\alpha_2\text{-}P^{V}_2W^{VI}_{17}O_{61}]^{10-}$ | S4C | 7.3 | 2.0; -7.3; -14.4 | $[\alpha_2\text{-}P^{V}_2W^{VI}_{17}O_{61}]^{10-}$ | S4D | 13 |
| $(NH_4)_{14}[NaP^{V}_5W^{VI}_{30}O_{110}]$ $P_5W_{30}$ | 3.5 | -10.1; -13.0 | -206.3; -208.3; -273.2; -285.8 | 99%[b] $[NaP^{V}_5W^{VI}_{30}O_{110}]^{14-}$ + 1%[b] $[\alpha\text{-}P^{V}_2W^{VI}_{18}O_{62}]^{6-}$ | 2A; 3 | 3.0 | -10.1; -13.0 | 99% $[NaP^{V}_5W^{VI}_{30}O_{110}]^{14-}$ + 1% $[\alpha\text{-}P^{V}_2W^{VI}_{18}O_{62}]^{6-}$ | 2B | 7.1 | 1.5; -10.1 | -204.4; -206.5; -272.2; -284.7 | $[NaP^{V}_5W^{VI}_{30}O_{110}]^{14-}$ | 2C; 3 | 6.8 | 1.5; -10.1 | $[NaP^{V}_5W^{VI}_{30}O_{110}]^{14-}$ | 2D | 14 |

[a]MHB – Mueller-Hinton broth, for more detailed information about composition see https://labmal.com/2019/11/20/mueller-hinton-agar-and-mueller-hinton-broth/

[b] The percentage is given based on the integration of $^{31}P$ signals.

## 3 NMR spectroscopy

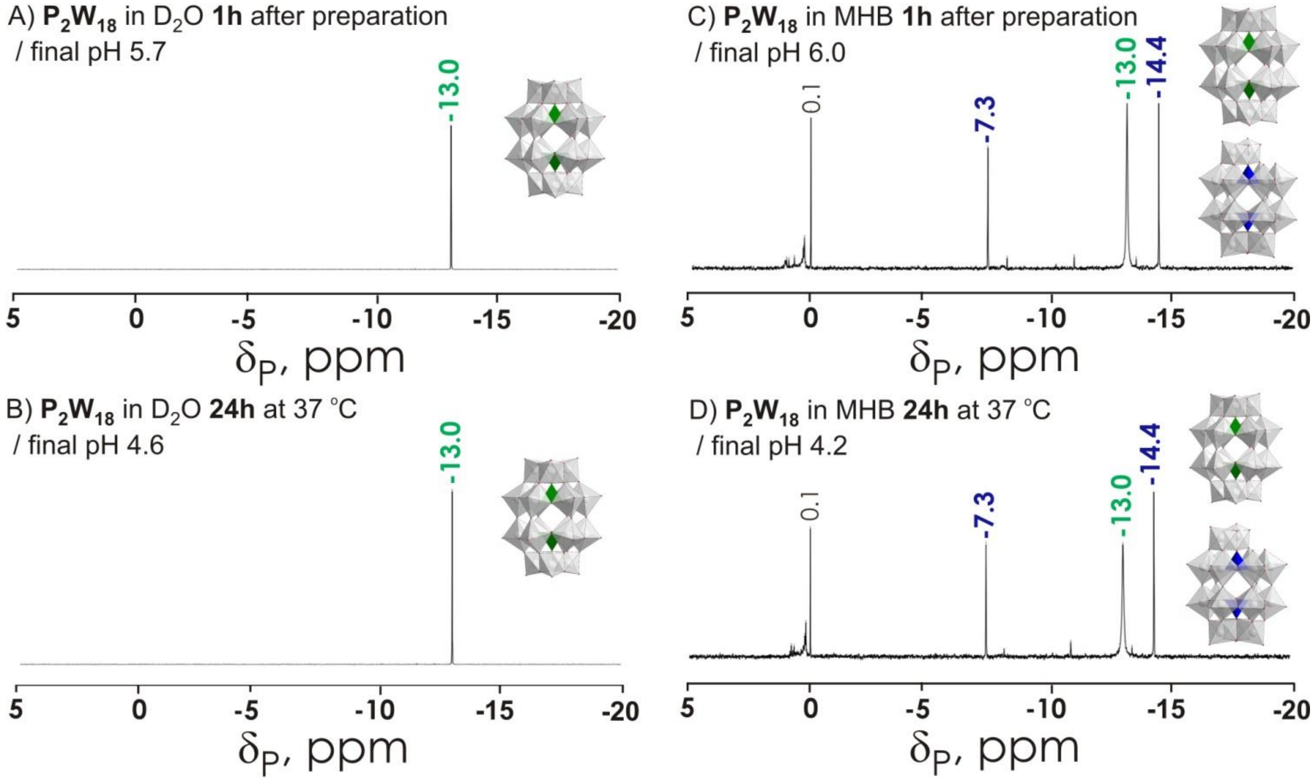


**Supplementary Figure S2.** $^{31}$P NMR spectra of 10 mM $K_6[\alpha\text{-}P^{V}_2W^{VI}_{18}O_{62}]\cdot P_2W_{18}$ solutions **A**) in $D_2O$ recorded approximately 1 h after preparation; **B**) in $D_2O$ recorded after incubation for 24 h at 37 °C; **C**) in Mueller-Hinton broth (MHB) recorded approximately 1 h after preparation; **D**) in Mueller-Hinton broth (MHB) recorded after incubation for 24 h at 37 °C. Signals at the region from 1 to 0 ppm in parts **C**) and **D**) correspond to free phosphates $H_xPO_4^{(3-x)-}$ (x = 0 – 3) from the medium. The signal at -13.0 ppm corresponds to 2 equivalent P ions in $P_2W_{18}$ shown in green; the signals at -7.3 ppm and -14.4 ppm correspond to two P ions in $[\alpha_2\text{-}P^{V}_2W^{VI}_{17}O_{61}]^{10-}$ $P_2W_{17}$ shown in blue. Based on the integration of the POTs' signals (signals of free phosphates were not taken into account), the concentration of $P_2W_{18}$ in MHB before incubation is 72% and after incubation 69%; the concentration of the hydrolysis product $P_2W_{17}$ is 27% before and 29% after incubation. Color code: {$WO_6$}, light grey; {$PO_4$}, green and blue; O, red.

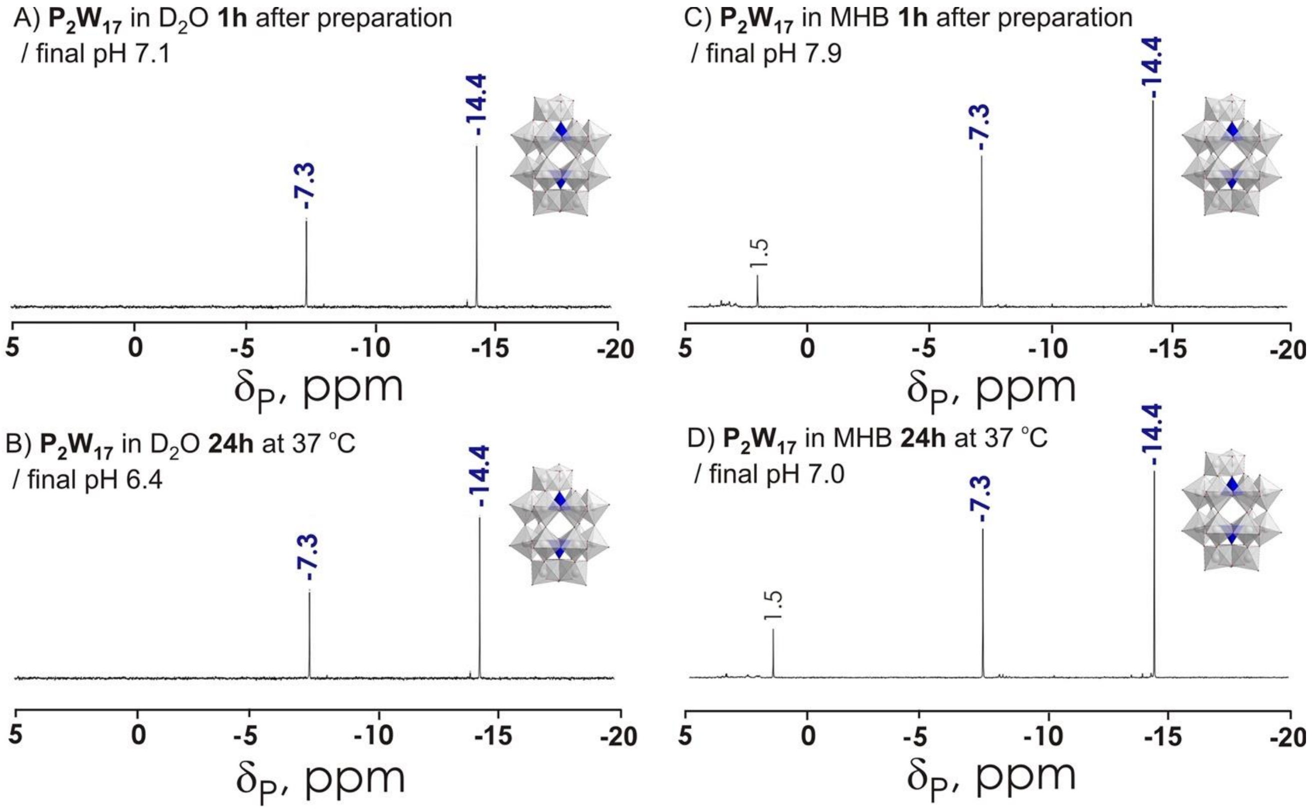


**Supplementary Figure S3.** $^{31}P$ NMR spectra of 10 mM $K_{10}[\alpha_2\text{-}P^{V}_2W^{VI}_{17}O_{61}]$ $P_2W_{17}$ solutions **A**) in $D_2O$ recorded approximately 1 h after preparation; **B**) in $D_2O$ recorded after incubation for 24 h at 37 °C; **C**) in Mueller-Hinton broth (MHB) recorded approximately 1 h after preparation; **D**) in Mueller-Hinton broth (MHB) recorded after incubation for 24 h at 37 °C. The signals at -7.3 ppm and -14.4 ppm correspond to two P ions in $P_2W_{17}$ shown in blue. Color code: {$WO_6$}, light grey; {$PO_4$}, blue; O, red.

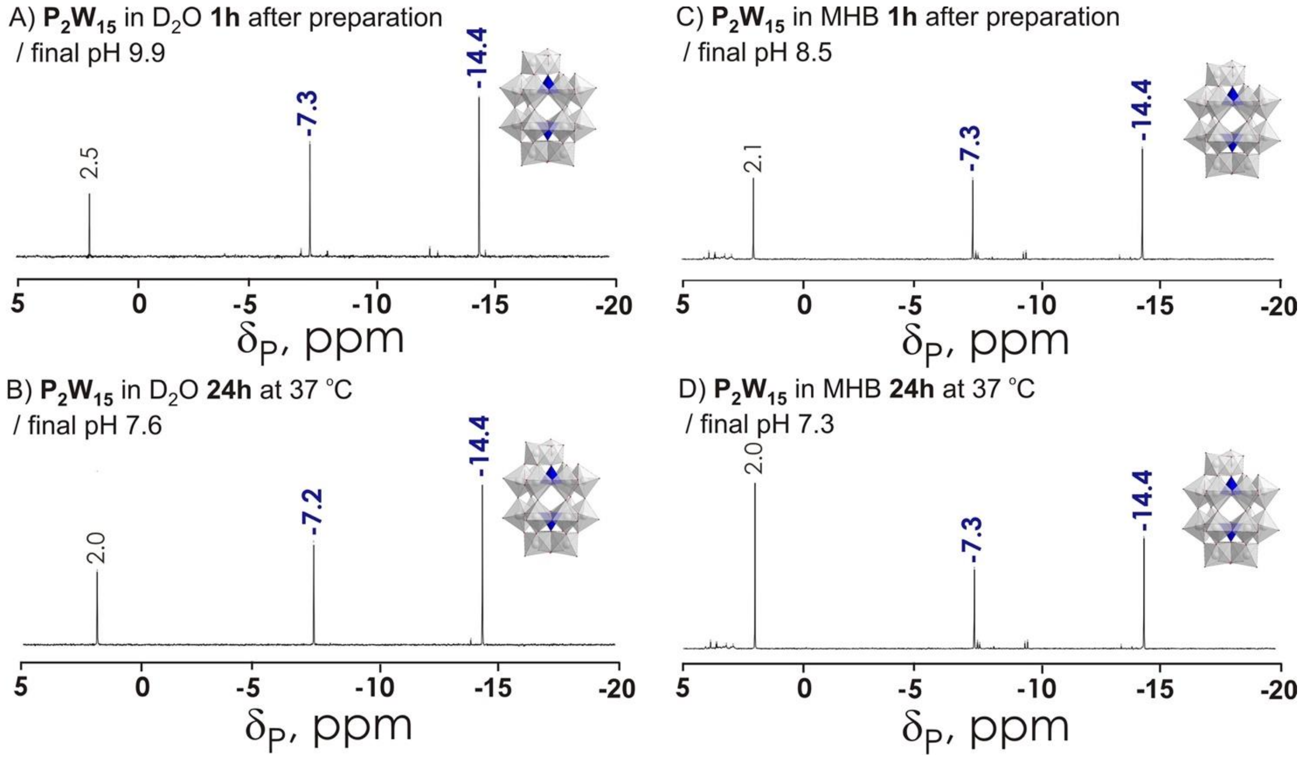


**Supplementary Figure S4.** $^{31}P$ NMR spectra of 10 mM $K_{12}[P^{V}_{2}W^{VI}_{15}O_{56}]$ $P_2W_{15}$ solutions **A**) in $D_2O$ recorded approximately 1 h after preparation; **B**) in $D_2O$ recorded after incubation for 24 h at 37 °C; **C**) in Mueller-Hinton broth (MHB) recorded approximately 1 h after preparation; **D**) in Mueller-Hinton broth (MHB) recorded after incubation for 24 h at 37 °C. The signals at -7.3 ppm and -14.4 ppm correspond to two P ions in $[\alpha_2\text{-}P^{V}_{2}W^{VI}_{17}O_{61}]^{10-}$ $P_2W_{17}$ shown in blue; signals at 2.5 corresponds to free phosphate phosphates $H_xPO_4^{(3-x)-}$ (x = 0 – 3). Color code: $\{WO_6\}$, light grey; $\{PO_4\}$, blue; O, red.

## 4 Inhibition zones

**Supplementary Table S4.** Inhibition zones (mm) produced by the tested polyoxotungstates; $K_{12}[P^{V}_2W^{VI}_{15}O_{56}]$ $24H_2O$ $P_2W_{15}$, $K_{10}[\alpha_2\text{-}P^{V}_2W^{VI}_{17}O_{61}]$ $20H_2O$ $P_2W_{17}$, $K_6[\alpha\text{-}P^{V}_2W^{VI}_{18}O_{62}]$ $14H_2O$ $P_2W_{18}$ and $(NH_4)_{14}[NaP^{V}_5W^{VI}_{30}O_{110}]\cdot 31H_2O$ $P_5W_{30}$ ranging from 100 to 500 μM.

| **Microorganisms*** | **$P_2W_{15}$ (μM)** | | | | | **$P_2W_{17}$ (μM)** | | | | | **$P_2W_{18}$ (μM)** | | | | | **$P_5W_{30}$ ((μM)** | | | | |
|---|---|---|---|---|---|---|---|---|---|---|---|---|---|---|---|---|---|---|---|---|
| | **100** | **200** | **300** | **400** | **500** | **100** | **200** | **300** | **400** | **500** | **100** | **200** | **300** | **400** | **500** | **100** | **200** | **300** | **400** | **500** |
| *A. baumannii* DSM 3007 | NI | NI | NI | NI | 9.0 ±0.7 | NI | NI | NI | NI | 8.5 ±0.5 | NI | NI | NI | 8.0 ± 0.7 | 8.5 ± 0.9 | 7.5 ± 1.9 | 6.8 ± 1.5 | 6.5 ± 1.0 | NI | NI |
| *A. baumannii* I73775 | NI | NI | NI | NI | NI | NI | NI | NI | NI | NI | NI | NI | NI | NI | NI | NI | 6.5 ± 1.0 | 8.3 ± 0.5 | 9.8 ± 0.5 | 10.5 ± 0.6 |
| *E. coli* DSM 1077 | NI | NI | NI | NI | NI | NI | NI | NI | NI | NI | NI | NI | NI | NI | NI | NI | NI | NI | NI | NI |
| *E. coli* I73194 | NI | NI | NI | NI | NI | NI | NI | NI | NI | NI | NI | NI | NI | NI | NI | 7.6 ± 0.5 | 7.8 ± 0.5 | 8.2 ± 0.3 | 8.9 ± 0.3 | 9.8 ± 0.5 |
| *K. pneumoniae* I70923/4 | NI | NI | NI | NI | NI | NI | NI | NI | NI | NI | NI | NI | NI | NI | NI | 6.5 ± 0.6 | 6.5 ± 0.6 | 8.0 ± 1.4 | 8.3 ± 1.3 | 8.3 ± 0.5 |
| *P. aeruginosa* C46281 | NI | 7.25 ±0.4 | 7.25 ±0.4 | 7.5± 0.9 | NI | NI | NI | NI | NI | NI | NI | NI | NI | 7.0± 0.7 | 7.0± 1.0 | 7.8± 1.8 | 6.5± 0.6 | 7.0± 0.8 | 7.5± 1.3 | 8.0 ± 0.8 |
| *S. aureus* ATCC 6538 | 13.0 ± 1.0 | 15.0 ± 0.5 | 15.0 ± 0.7 | 15.5 ± 0.5 | 15.0± 0.0 | 12.5 ± 0.5 | 14.5 ± 0.5 | 14.0± 0.0 | 14.0± 0.0 | 16.0± 0.0 | 11.5± 0.5 | 14.0± 0.0 | 15.0 ± 0.0 | 15.0± 0.0 | 15.5± 0.5 | 13.0± 1.4 | 15.5± 2.1 | 15.5± 0.7 | 16.0 ±0.0 | 16.0 ± 5.7 |

| | | | | | | | | | | | | | | | | | | | | |
|---|---|---|---|---|---|---|---|---|---|---|---|---|---|---|---|---|---|---|---|---|
| *S. pneumoniae* D39 | NI | NI | NI | 8.25 ± 1.6 | 9.5± 1.1 | NI | 8.0± 1.0 | 8.75±1.3 | 10.0± 1.0 | 10.5± 1.5 | 7.5± 0.9 | 8.0±1.6 | 9.25 ± 1.3 | 10± 1.0 | 10.5± 1.1 | 8.5± 0.6 | 9.5± 0.6 | 10.5± 0.6 | 11.0 ± 0.0 | 11.5 ± 0.6 |

*Data represent the mean±standard deviation. NI- No Inhibition

## 5 References


**1.** Chen, K., Yu, Q., Liu, Y. & Yin, P. (2021) Bacterial hyperpolarization modulated by polyoxometalates for solutions of antibiotic resistance. *J. Inorg. Biochem.* 220, 111463.

2. Hill, C.L., Weeks, M.S., Schinazi, R.F. (1990). Anti-HIV-1 activity, toxicity, and stability studies of representative structural families of polyoxometalates. J. Med. Chem. 33(10), 2767–2772.

3. Zhou, Y.W., Zheng, L., Han, F., Zhang, G.J., Mac, Y., Yao, J.N., Keita, B., de Oliveira, P., Nadjo, L. (2011). Inhibition of amyloid-beta protein fibrillization upon interaction with polyoxometalates nanoclusters. colloids surf. a physicochem. Eng. Aspects, 375, 97-101.

4. Fu, L., Gao, H., Yan, M., Li, S., Li, X., Dai, Z., Liu, S. (2015). Polyoxometalate-based organic–inorganic hybrids as antitumor drugs. Small, 11, 2938-2945.

5. Du, J., Cao, M.D., Feng, S.L., Su, F., Sang, X.J., Zhang, L.C., You, W.S., Yang, M., Zhu, Z.M. (2017). Two new Preyssler-type polyoxometalate-based coordination polymers and their application in horseradish peroxidase immobilization. Chem. Eur. J. 23, 14614.

6. Gumerova, N.I., Al-Sayed, E., Krivosudsky, L., Cipic-Paljetak, H., Verbanac, D., Rompel, A. (2018). Antibacterial activity of polyoxometalates against *Moraxella catarrhalis*. Front. Chem. 6: 336.

7. Haider, A., Zarschler, K., Joshi, S.A., Smith, R.M., Lin, Z.G., Mougharbel, A.S., Herzog, U., Müller, C.E., Stephan, H., Kortz, U. (2018). Preyssler-Pope-Jeannin polyanions $[NaP_5W_{30}O_{110}]^{14-}$ and $[AgP_5W_{30}O_{110}]^{14-}$: Microwave-assisted synthesis, structure, and biological activity. Z. Anorg. Allg. Chem. 644, 752-758.

8. Pimpao, C., da Silva, I.V., Mosca, A.F., Pinho, J.O., Gaspar, M.M., Gumerova, N.I., Rompel, A., Aureliano, M., Soveral, G. (2020). The Aquaporin-3-inhibiting potential of polyoxotungstates. Int. J. Mol. Sci. 21(7): 2467.

9. Razavi, S.F., Bamoharram, F.F., Hashemi, T., Shahrokhabadi, K., Davoodnia, A. (2020). Nanolipid-loaded Preyssler polyoxometalate: synthesis, characterization and in vitro inhibitory effects on HepG2 tumor cells. Toxicol. In Vitro 68: 104917.

10. Xu, Z., Chen, K., Li, M., Hu, C., Yin, P. (2020). Sustained release of $Ag^+$ confined inside polyoxometalates for long-lasting bacterial resistance. Chem. Commun. 56: 5287-5290.

11. Chen, K., Yu, Q., Liu, Y., Yin, P. (2021). Bacterial hyperpolarization modulated by polyoxometalates for solutions of antibiotic resistance. J. Inorg. Biochem. 220: 111463.

12. Aureliano, M., Fraqueza, G., Gumerova, N.I., Rompel, A., Cordoba-Granados, J.J., Berrocal, M. and Mata, A. M. (2021). Comparison of SERCA and PMCA inhibition potential of polyoxotungstates, XXI SPB Congress Book, pag. 13, ISBN: 978-972-778-215-4, Universidade de Évora Ed., Évora, October 14-14.

13. Contant, R., Klemperer, W. G., and Yaghi, O. (2007). “Potassium octadecatungstodiphosphates(V) and related lacunary compounds,” in *Inorganic Syntheses*, ed A. P. Ginsberg (New York, NY: John Wiley & Sons, Inc.), 104–111.

14. Jeannin, Y., Martin-Frere, J., Choi, D. J., and Pope, M. T. (2007). “The sodium pentaphosphato(V)-triacontatungstate anion isolated as the ammonium salt,” in *Inorganic Syntheses*, ed A. P. Ginsberg (New York, NY: John Wiley & Sons, Inc.), 115–118.
